# Assisted Reproductive Technology and Breech Delivery: A Nationwide Cohort Study in Singleton Pregnancies

**DOI:** 10.3390/jpm13071144

**Published:** 2023-07-16

**Authors:** Ambrogio P. Londero, Claudia Massarotti, Anjeza Xholli, Arrigo Fruscalzo, Angelo Cagnacci

**Affiliations:** 1 Department of Neuroscience, Rehabilitation, Ophthalmology, Genetics, Maternal and Infant Health (DiNOGMI), University of Genoa, 16132 Genova, Italy; 2 Obstetrics and Gynecology Unit, IRCCS Istituto Giannina Gaslini, 16147 Genova, Italy; 3 Academic Unit of Obstetrics and Gynecology, IRCCS Ospedale San Martino, 16132 Genoa, Italy; 4 Clinic of Obstetrics and Gynecology, HFR Fribourg, 1700 Fribourg, Switzerland

**Keywords:** assisted reproduction technologies, ART, in vitro fertilization, IVF, intracytoplasmic sperm injection, ICSI, subfertility treatments, fertility-enhancing drugs, intrauterine insemination, breech presentation

## Abstract

Since essential factors have changed in recent years in assisted reproduction technologies (ARTs), this study reassessed the association between ART and breech presentation. We primarily aimed to estimate the correlation between ART and breech at delivery. Secondary purposes were to evaluate the correlation between other subfertility treatments (OSTs) and breech and to assess possible confounding factors and temporal trends. This study investigated the 31,692,729 live birth certificates from US states and territories in the 2009–2020 period. The inclusion criteria were singleton births reporting the method of conception and the presentation at delivery. The outcome was the breech presentation at delivery, while the primary exposure was ART, the secondary exposure was OST, and the potential confounding factors from the literature were considered. ART (OR 2.32 CI.95 2.23–2.41) and OST (OR 1.79 CI.95 1.71–1.87) were independent and significant risk factors for breech at delivery (*p* < 0.001). This study confirmed breech presentation risk factors maternal age, nulliparity, tobacco smoke, a previous cesarean delivery (CD), neonatal female sex, gestational age, and birth weight. Black race and Hispanic origin were verified to be protective factors. We found breech prevalence among ART and OST to be stable during the study period. Meanwhile, newborn birth weight was increased, and the gap between breech and other presentations in ART was reduced. Our results indicate that singleton pregnancies conceived by ART or OST were associated with a higher risk of breech at delivery. Well-known risk factors for the breech presentation were also confirmed. Some of these factors can be modified by implementing interventions to reduce their prevalence (e.g., tobacco smoke and previous CD).

## 1. Introduction

Since the introduction in the 1970s of assisted reproduction technology (ART) in the human species, many improvements have been implemented [1,2]. In recent decades, in vitro fertilization (IVF) and intracytoplasmic sperm injection (ICSI) have been increasingly associated with frozen embryo transfer (FET) following the freeze-all concept [3]. Many factors have contributed to this adoption, such as improving the in vitro culture techniques and introducing vitrification procedures [3,4].

Although in the past, singleton infants conceived with ART showed a higher hazard for low birth weight and prematurity, the introduction of the freeze-all concept decreased some negative perinatal outcomes such as the low birth weight [5,6]. However, other adverse effects increased, such as the risk of hypertensive disorders of pregnancy, macrosomia, and postpartum hemorrhage [7,8].

The breech presentation is a familiar condition in obstetrical practice, accounting for approximately 3–8% of singleton fetuses at delivery [9,10,11]. Moreover, the breech presentation in labor is a demanding vaginal delivery condition associated with adverse outcomes [11]. Since the Term Breech Trial exhibited reduced perinatal mortality and short-term morbidity associated with a planned cesarean delivery, the breech presentation has become a usual indication for a cesarean delivery [11,12,13].

ART was previously associated with breech presentation [14,15,16]. However, in the literature, including an old series of ART pregnancies, an increased prevalence of breech presentation among ART fetuses was found to be mediated by lower parity and shorter gestational length [2]. Among the priority objectives of modern obstetrics is the reduction in cesarean deliveries (CDs), since the expanded number of primary CDs is conducive to an increase in maternal morbidity and mortality [17,18]. The progressive abandonment of the breech vaginal birth increased the interest in the risk factors associated with the breech presentation to contain breech prevalence and consequently reduce the cesarean section rate. Furthermore, since many factors have changed in recent years in ART praxis, this study aimed to reassess the association between ART and breech presentation. Our main objective was to estimate the correlation between ART and breech presentation at delivery. The secondary aim was to evaluate the correlation between other subfertility treatments (OSTs) and breech presentation at delivery. Other secondary objectives were to assess possible confounding factors and the temporal trends.

## 2. Materials and Methods

### 2.1. Design, Setting, and Sample

**Figure 1:**
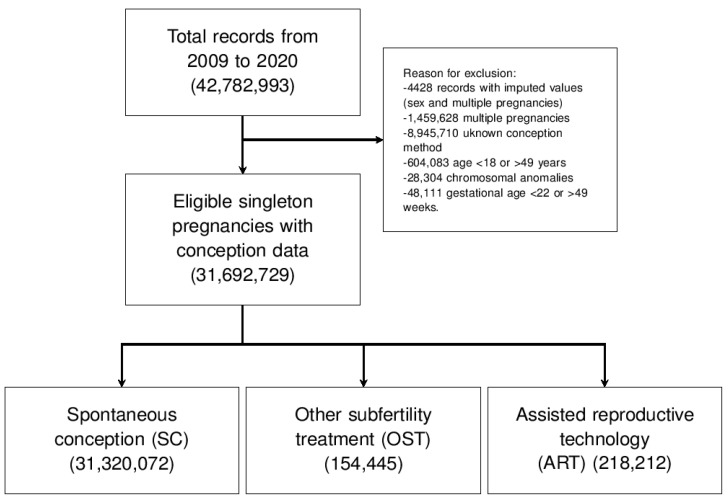
The study flow diagram.

In this cross-sectional retrospective study, we used the US National Center for Health Statistics birth certificate data that are part of the National Vital Statistics System [19]. We considered the period from 2009 to 2020 (including States and Territories). Data on the mode of conception were reported from 2009 with full coverage starting from 2016 [20,21]. This study employed data from 31,692,729 singleton births that reported the method of conception and the use of ART (Figure 1). The designated period and registry were chosen due to the availability of the necessary data and the growing use of the FET.

Pregnancies were selected according to the subsequent inclusion and exclusion criteria. We included all consecutive singleton pregnancies with information about the mode of conception and maternal age at delivery between 18 and 49 years old. We considered the following as exclusion criteria: women older than 49 years old or younger than 18 years old, records with imputed values for sex or multiple pregnancies, multiple pregnancies, gestational age below 22 or above 49 weeks of gestation, no data about the mode of conception, and chromosomal anomalies. In Figure 1, we show the population selection flowchart.

While preparing the manuscript, we observed the Strengthening the Reporting of Observational Studies in Epidemiology (STROBE) statement guidelines for observational studies (http://www.strobe-statement.org/, accessed on 1st May 2023) [22]. All data used for these analyses were de-identified and publicly available. For this reason, the local Ethics Committee’s approval for this study was not required. Moreover, we executed this study according to the principles of the Helsinki declaration.

### 2.2. Variables

From the original datasets, the following variables were extracted: maternal age, parity, race, pre-pregnancy body mass index (BMI), fetal presentation at delivery, mode of delivery, previous CD, multiple pregnancies, multiple pregnancies imputation label, neonatal sex, neonatal sex imputation label, gestational age at delivery, neonatal weight, chromosomal anomalies, and tobacco smoke. The primary outcome was the presentation at birth. Type of conception was used as the primary explanatory variable [2]. The type of conception was categorized into three levels: spontaneous conception, OST (non-ART treatment, including fertility-enhancing drugs and intrauterine insemination), and ART (including in vitro fertilization, intracytoplasmic sperm injection, and gamete and zygote intrafallopian transfer procedures) [23,24,25]. The potential confounding factors were derived from the literature and included the following: maternal age, parity, previous CD, tobacco smoke, gestational age, race (Black race and Hispanic origin), neonatal sex, and neonatal weight [11,26,27,28,29,30,31,32,33,34,35,36,37,38,39]. Maternal age was considered a continuous variable. Parity was dichotomized in nulliparous versus parous women. The previous CD was classified as present (any previous CD) versus no previous CD. Tobacco smoke was classified as the presence of smoking habits in any of the three pregnancy trimesters. Gestational age was mainly established on completed weeks of gestation from the date of the last menstrual period [24]. Race was categorized according to the following categories: single black race and Hispanic origin. Neonatal sex was coded as male or female sex categories. Neonatal weight was recorded in grams and the relative multiple of the median (MoM) was calculated using the Fenton post-natal standards (utilizing neonatal weight, sex, and gestational age at delivery) [40]. In particular, neonatal weight MoM was calculated as follows: neonatal weight/50th centile of neonatal weight at the same gestational age-adjusted per neonatal sex [39,40]. Fetal presentation at delivery was recorded as cephalic, breech, other presentation, and unknown. The mode of delivery was categorized as spontaneous, forceps, vacuum, cesarean, and unknown. The unknown values were considered missing (“NA”) in all other variables unless otherwise specified. A detailed explanation of the variables is available on the following website: https://www.cdc.gov/nchs/data_access/Vitalstatsonline.htm#Tools (accessed on 2 December 2021).

### 2.3. Data Analysis

All investigations were performed using R software (version 4.1.3) [41]. The probability values were 2-sided, and we assumed a *p*-value < 0.05 to be statistically significant. The normality of continuous variables was evaluated using the Kolmogorov–Smirnov test. We show parametric continuous variables as mean ± standard deviation and non-parametric ones as the median and interquartile range (IQR). We display dichotomous or polychotomous variables as percentages and absolute values, excluding missing values (“NA”) from the denominator (unless otherwise defined). We show the logistic regression results as odds ratio (OR) and 95% confidence interval (CI.95). We analyzed categorical variables (dichotomous or polychotomous) employing the chi-square test or the Fisher exact test. Meanwhile, we analyzed continuous variables using the Wilcoxon test (non-parametric variables) or the *t*-test (parametric variables). We also conducted a logistic regression analysis considering fetal presentation as a dependent variable and mode of conception (i.e., ART) as the primary explanatory variable (independent). We also considered all possible confounding factors derived from the literature and available in the dataset as independent variables. We carried all potential predictive factors (*p* < 0.05) from univariate analysis in the multivariate model. The initial multivariate model incorporated all variables and their interactions, and when interactions turned out to be non-significant, the estimation without interaction model was employed.

## 3. Results

### 3.1. Population Characteristics

The total number of singleton pregnancies to comply with the inclusion criteria was 31,692,729. The median maternal age was 29 years (IQR 24–33). The median pre-gestational BMI was 25 kg/m^2^ (IQR 22–30), and 31.24% of women were nulliparous. The majority were white only (74.73%), 14.92% were black only, and 25.55% were of Hispanic origin. A positive history of tobacco smoke was present in 7.35% of cases, and the history of a previous CD was present in 15.12% of cases. In the majority of cases, an SC of 98.82% was recorded (31,320,072); in 0.49%, an OST of 154,445; and in 0.69%, an ART of 218,212.

The median gestational age at delivery was 39 weeks (IQR 38–40), the median birth weight was 3320 g (IQR 3005–3647), and the median birth weight MoM was 0.99 (IQR 0.90–1.08). Most (94.17%) fetuses at delivery were in cephalic presentation, and 3.00% were in breech presentation. Spontaneous vaginal birth delivery was observed in 65.67% of cases and CD in 31.04%.

### 3.2. Population Characteristics and Mode of Conception

**Figure 2:**
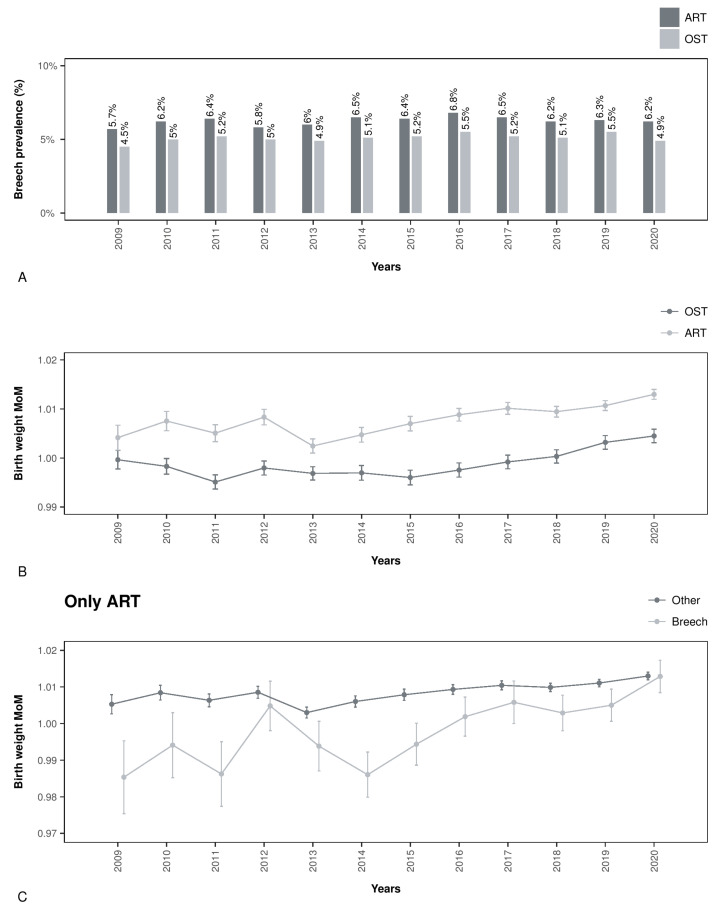
Panel (**A**) shows the time trend in breech presentation prevalence among OST (154,445 singleton pregnancies) and ART (218,212 singleton pregnancies). Panel (**B**) shows the birth weight MoM trend among the study years in OST (154,395 singleton pregnancies) and ART (218,122 singleton pregnancies). Panel (**C**) shows the birth weight MoM time trend in 218,122 ART pregnancies stratified per fetal presentation at delivery (breech vs. others) among the study years.

Table 1 shows the differences between methods of conception in the population characteristics and breech presentation at delivery. In particular, the breech presentation was 2.97% among SC, 5.12% among OST, and 6.32% among ART (all the differences were statistically significant). Figure 2A shows that the breech prevalence was stable over the years in OST and ART groups. Moreover, the high breech prevalence contributes to the high CD prevalence among ART pregnancies (47.73%). And the CD prevalence was also stable over the years (Figure S1). Furthermore, birth weight and birth weight MoM were significantly higher in the ART group than in the other groups (*p* < 0.05). Figure 2B shows a trend of the birth weight MoMs increasing over the years in OST and ART groups while keeping ART at significantly higher levels than OST. Moreover, Figure 2C shows the differences in birth weight MoMs between breech and other presentations in the ART group. We found an increasing birth weight MoM value in breech-presenting fetuses among ART, arriving at the same values as other presentations in 2020 (Figure 2C). A lower-weight MoM value in breech-presenting fetuses was also found in the OST group (Figure S2).

### 3.3. ART as a Risk Factor for Breech Presentation and Other Known Risk Factors

**Figure 3:**
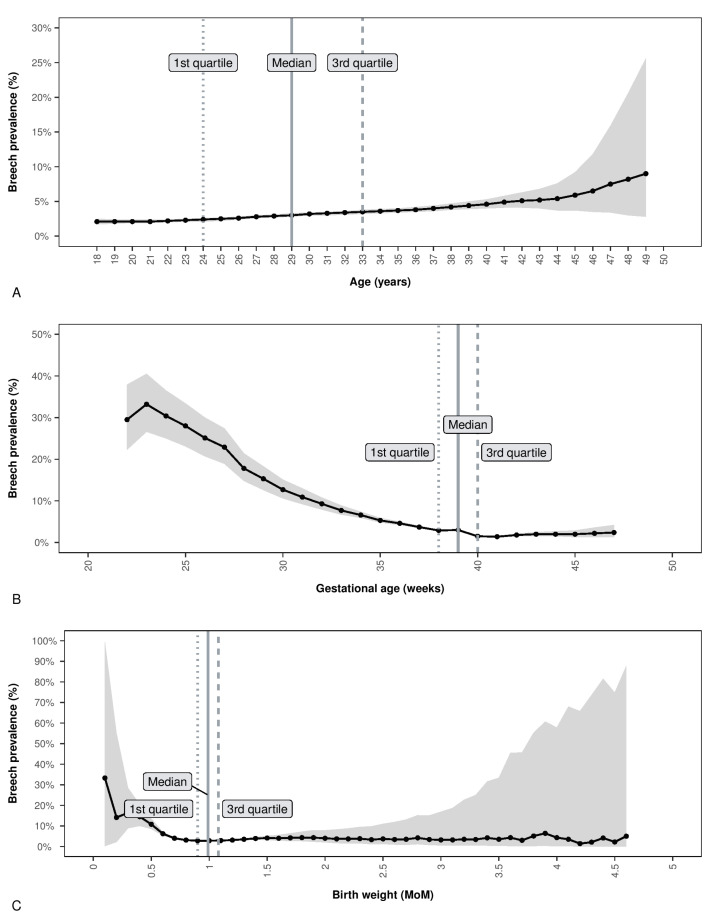
This figure shows the risk of breech presentation at delivery according to maternal age, gestational age, and birth weight MoM at delivery (the gray area represents the 95% confidence interval). Panel (**A**) shows the risk of breech presentation in percentage for every age in the whole cohort of 31,692,729 singleton deliveries. Panel (**B**) shows the risk of breech presentation in percentage for each gestational week in the entire cohort of 31,692,729 singleton deliveries. Panel (**C**) shows the risk of breech presentation in percentage for every birth weight MoM in the cohort of 31,677,863 (14,866 are NA) singleton deliveries.

ART was a significant risk factor for breech presentation at delivery OR 2.2 (CI.95 2.16–2.23) (Table 2). Other risk factors were maternal age, gestational age, and newborn birth weight MoMs. All these three continuous variables were found to be non-parametric in the studied population. Figure 3A shows a progressively increasing risk with increasing maternal age, with the highest risk above the third quartile of the distribution (OR 1.49, CI.95 1.48–1.49, *p* < 0.001) (Table 2). Figure 3B shows a progressively decreasing risk with increasing gestational age at delivery, with the lowest risk above the third quartile of the distribution (OR 0.48, CI.95 0.48–0.49, *p* < 0.001) (Table 2). Meanwhile, newborn birth weight MoM had a U-shaped risk, showing an increased risk below the first and above the third quartile of the distribution (respectively, OR 1.20 CI.95 1.20–1.21 and OR 1.13 CI.95 1.13–1.14) (*p* < 0.001) (Table 2). The multivariate logistic regression analysis is also presented in Table 2 and Table S1. Both ART and OST were also independent risk factors for breech presentation at delivery after adjusting for the possible confounding factors (including maternal, parity, and gestational age at birth) (Table 2 and Table S1).

## 4. Discussion

### 4.1. Key Results

We found ART and OST to be independent and significant risk factors for breech presentation at delivery. Our analysis confirmed risk factors for breech presentation to be maternal age, nulliparity, tobacco smoke, previous CD, neonatal female sex, gestational age, and neonatal birth weight. Black race and Hispanic origin were confirmed to be protective factors. We further found breech prevalence among ART and OST to be stable during the study period. Meanwhile, newborn birth weight MoM was increased during the study period, and the gap between breech and other presentations in ART was reduced to a non-significant difference.

### 4.2. Interpretation and Comparison with the Literature

The literature is controversial about the association between ART and breech presentation at delivery. Previously published small observational studies have documented a raised risk of the breech presentation associated with ART [15,16,42,43], but these studies were underpowered for taking into account potentially confounding factors. Meanwhile, a large cohort study found that the increased risk of breech presentation in ART pregnancies was mediated by maternal age, maternal parity, and gestational length [2]. However, all these data were based on historical datasets earlier than 2006 [2,43]. Afterward, many new technologies were introduced in ART. Our data found ART and OST to be independent factors for breech presentation in a series of pregnancies between 2009 and 2020. And this effect was not fully mediated by gestational age or parity, as previously found by Romundstad and coworkers [2].

Multiple hypotheses can explain our findings. First, as both OST and ART are associated with breech presentation, some factors related to subfertility are probably associated with the risk of breech presentation at delivery independently of gestational age, parity, and the other factors accounted for in this study. This hypothesis is corroborated by previous data where adverse pregnancy outcomes were attributed to factors associated with infertility rather than to elements linked to ART [44]. Second, it is known that ART pregnancies present differences in the placenta than spontaneously conceived pregnancies. ART was significantly associated with an increased placental index [45], accelerated villous maturation, and increased distal villous hypoplasia [46]. These findings are probably both manifestations of a compensatory response by the placenta to improve its transport capacity in the specific environment of in vitro fertilization [45,46]. In particular, the increased placental index indicates more volume of the placenta per volume of the fetus, which can impair fetal movements. Similarly, an increased placental index was hypothesized to impair fetal movements and favor a higher prevalence of breech in female fetuses [11]. Moreover, this study confirmed the literature finding that female fetal gender is an independent risk factor for breech presentation at delivery [11]. We further confirmed older maternal age as a risk factor for breech presentation [11,27,31]. Early gestational age was also confirmed as a significant risk factor [2,11,26,28,29,35,38]. Nulliparity was also verified to be a significant risk factor for breech presentation at delivery [2,11,26,28,35,38]. Our analysis also confirmed tobacco smoke to be a significant risk factor for breech presentation independently from neonatal birth weight MoM [38]. A previous CD was also confirmed to be a significant risk factor for breech presentation at delivery [33,37]. Low neonatal weight was previously found to be associated with breech presentation at delivery [11,35,36]. We found neonatal weight MoMs to have a U-shaped risk for breech presentation at delivery. We confirmed low-neonatal-weight MoMs to be associated with breech at delivery, and even if to a minor extent, we also found high-neonatal-weight MoMs to be associated with breech presentation.

In the US population, the number of FETs on the total number of embryo transfers (ET) increased from 31.5% in 2014 to 44.1% in 2019 in cycles with the patient’s own oocytes alone (SART data—https://www.sartcorsonline.com—accessed on 12 April 2022). The neonatal birthweights also increased, coherently with the literature linking FET to large for gestational age newborns [47]. Instead, there was no significant decrease in the breech prevalence in the ART group, confirming infertility/ART as a predictor for breech presentation, regardless of the incidence of low birthweight.

Additionally, our data confirm a protective role for the Black race and Hispanic origin [30].

In previously published data, Romundstad and coworkers and Kallen and coworkers found a significant decreasing trend in the years among the ART pregnancies’ CD prevalence [2,48]. In a different setting and temporal period, we found a stable prevalence of CD in ART and OST cephalic pregnancies that is constantly higher than in SC cephalic pregnancies (Figure S2). The same trend was observed in breech pregnancies; however, breech presentation is known to increase negative perinatal outcomes, and after the Term Breech Trial results, planned cesarean delivery has gained popularity [12,13,49]. Although other groups reported data about the CD time trend in ART, to the best of our knowledge, this is the first study reporting the time trend of CD risk in OST according to breech and cephalic presentations [2,48].

Our results confirmed disparities in ART and OST utilization with low prevalence among Black-only race and Hispanic origin [21,50].

### 4.3. Strengths and Weaknesses

The major strength of this investigation was its population-based design, and this attribute implies the inclusion of a large number of singleton pregnancies. Furthermore, a vast corpus of information was available, comprising most of the known potentially confounding factors. However, as it is specific for large population registries, data on ART procedures lack granularity. In particular, details about controlled ovarian stimulation and day of ET were missing, and it is not known if the pregnancies resulted from ET or FET. This last information can be inferred by the national SART registry: the number of FETs steadily increased in the last decade, but there was no variation in breech prevalence among ART pregnancies. Some other information on factors known to be associated with both ART and breech presentation were missing, such as uterine malformations [51]. However, taking these additional characteristics into account is not likely to substantially influence the noticed effects, because of their relatively low prevalence and the equal distribution between subfertile and fertile patients [52]. Another piece of missing information was the presence of leiomyoma that was previously associated with both ART and breech presentation [53]. However, this correlation is probably mild, and the literature presents contradictory data [53,54]. Furthermore, fibroids are more present in the Black race, which is a protective factor against breech presentation [55]. Other details that were missing were fundal placenta position, the amniotic fluid amount, and familial predisposition, which are known to be related to breech presentation [34,38]. But it is unlikely that these factors also correlate with subfertility and, therefore, would not have influenced our results.

### 4.4. Generalizability, Relevance of the Findings, and Unanswered Questions

The generalizability of these results is based on four topics: the analysis of a large nationwide cohort, the investigation of a near period, the heterogeneity of the population included, and the verification of known risk factors (for breech presentation) also found in different settings. These points mean that these results are readily applicable also in other contexts. However, not all the results are equally generalizable. For example, the CD rate and the use of ART and OST change according to local management and the policies of the different health systems, and this issue limits the generalizability of our findings.

These data suggest that there is still space to reduce some of the risk factors associated with breech presentation and thus reduce the CD rate. For example, interventions aimed at reducing tobacco smoke in pregnancy or preventing primary CD can also favor the reduction in breech presentation. Furthermore, there is probably room for a further decrease in CDs favoring a greater diffusion for the version for external maneuvers from breech to cephalic.

However, studies are needed to demonstrate whether these interventions can actually be cost-effective. Furthermore, the connection between breech presentation and ART is not yet evident; further studies to better understand these mechanisms would lead to benefits. Again, additional development of ART and OST techniques could further reduce the differences observed between pregnancies obtained through these routes and those conceived spontaneously.

## 5. Conclusions

In summary, our results reveal that singleton pregnancies conceived by ART or OST were associated with a higher risk of breech presentation at delivery. We confirmed well-known risk factors for breech presentation at delivery, such as maternal age, nulliparity, tobacco smoke, previous CD, neonatal female sex, gestational age, neonatal birth weight, and protective factors such as Black race and Hispanic origin. Some of these factors can be modified by implementing further interventions to reduce their prevalence (e.g., tobacco smoke and previous CD). Furthermore, the CD rate is at the highest level among ART and OST singleton pregnancies, and there is probably a margin to lower it.

## Figures and Tables

**Table 1:** Population characteristics and differences between spontaneous conception (SC), other subfertility treatments (OST), and assisted reproductive technologies (ARTs).

	SC (31,320,072)	OST (154,445)	ART (218,212)	*p*
Patient characteristics				
Maternal age (years)	28.00 (24.00–33.00)	32.00 (29.00–36.00)	35.00 (32.00–39.00)	1, 2, 3
Pre-gestational BMI (kg/m^2^)	25.20 (22.00–30.10)	25.50 (22.10–31.10)	24.40 (21.70–28.80)	1, 2, 3
Nulliparity	31.11% (9,742,763/31,320,072)	45.24% (69,878/154,445)	41.03% (89,533/218,212)	1, 2, 3
Black-only race	15.04% (4,709,420/31,320,072)	4.61% (7116/154,445)	5.26% (11,481/218,212)	1, 2, 3
Hispanic origin	25.75% (7,998,917/31,063,364)	8.26% (12,659/153,317)	8.35% (17,729/212,222)	1, 2
Tobacco smoke	7.42% (2,280,828/30,727,907)	1.40% (2139/152,715)	0.52% (1129/216,231)	1, 2, 3
Previous CD	15.14% (4,743,336/31,320,072)	11.65% (17,988/154,445)	14.01% (30,578/218,212)	1, 2, 3
Gestational age (weeks)	39.00 (38.00–40.00)	39.00 (38.00–40.00)	39.00 (38.00–40.00)	1, 2, 3
Neonatal female sex	48.81% (15,286,830/31,320,072)	48.83% (75,413/154,445)	48.88% (106,663/218,212)	NS
Birth weight (grams)	3323.00 (3005.00–3646.00)	3326.00 (2990.00–3657.00)	3340.00 (2984.00–3671.00)	1, 2, 3
Birth weight (MoM)	0.99 (0.89–1.08)	0.99 (0.90–1.08)	1.00 (0.90–1.10)	1, 2, 3
Pregnancy and labor characteristics				
Fetal presentation				
Cephalic	94.19% (29,500,700/31,320,072)	92.99% (143,625/154,445)	91.46% (199,568/218,212)	1, 2, 3
Breech	2.97% (930,310/31,320,072)	5.12% (7909/154,445)	6.32% (13,790/218,212)	1, 2, 3
Other	1.45% (452,897/31,320,072)	1.18% (1826/154,445)	1.27% (2769/218,212)	1, 2, 3
Unknown	1.39% (436,165/31,320,072)	0.70% (1085/154,445)	0.96% (2085/218,212)	1, 2, 3
Mode of delivery				
Spontaneous	65.85% (20,623,069/31,320,072)	55.69% (86,010/154,445)	46.93% (10,2413/218,212)	1, 2, 3
Forceps	0.56% (174,834/31,320,072)	1.15% (1778/154,445)	1.20% (2609/218,212)	1, 2
Vacuum	2.67% (835,247/31,320,072)	3.86% (5955/154,445)	4.11% (8978/218,212)	1, 2, 3
Cesarean	30.88% (9,672,623/31,320,072)	39.27% (60,658/154,445)	47.73% (104,156/218,212)	1, 2, 3
Unknown	0.05% (14,299/31,320,072)	0.03% (44/154,445)	0.03% (56/218,212)	1, 2

Acronyms: SC = spontaneous conception; OST = other subfertility treatments; ART = assisted reproductive technologies; BMI = body mass index; CD = cesarean delivery; MoM = multiple of the median. Differences statistically significant (*p* < 0.05): (1) SC vs. OST; (2) SC vs. ART; (3) OST vs. ART.

**Table 2:** This table shows a logistic regression analysis that considers breech presentation at delivery as the dependent variable and the possible known risk factors as independent variables. Univariate and multivariate (*) analysis. The complete multivariate model with interaction terms is shown in Table S1.

	OR (CI.95)	*p*	OR (CI.95) (*) (¶)	*p* (*) (¶)
Factors associated with breech				
Maternal age >33 years	1.49 (1.48–1.49)	<0.001	1.39 (1.38–1.41)	<0.001
Nulliparity	1.36 (1.35–1.36)	<0.001	1.79 (1.78–1.81)	<0.001
Black only race	0.77 (0.76–0.77)	<0.001	0.75 (0.74–0.76)	<0.001
Hispanic origin (†)	0.82 (0.82–0.83)	<0.001	0.81 (0.8–0.82)	<0.001
Tobacco smoke (‡)	1.13 (1.12–1.13)	<0.001	1.18 (1.16–1.2)	<0.001
Previous CD	1.28 (1.28–1.29)	<0.001	1.53 (1.51–1.55)	<0.001
Neonatal female sex	1.15 (1.14–1.15)	<0.001	1.2 (1.19–1.21)	<0.001
Gestational age > 40 weeks	0.48 (0.48–0.49)	<0.001	0.41 (0.4–0.42)	<0.001
Birth weight (MoM) (§)				
<0.90 MoM	1.2 (1.2–1.21)	<0.001	1.47 (1.46–1.49)	<0.001
0.90–1.07 MoM	Reference	1.000	Reference	1.000
>1.07 MoM	1.13 (1.13–1.14)	<0.001	1.06 (1.05–1.07)	<0.001
OST	1.75 (1.71–1.79)	<0.001	1.79 (1.71–1.87)	<0.001
ART	2.2 (2.16–2.23)	<0.001	2.32 (2.23–2.41)	<0.001

Missing values: (†): 263826; (‡): 595876; (§): 14866; (¶): 851640. Acronyms: OST = other subfertility treatments; ART = assisted reproductive technologies; BMI = body mass index; CD = cesarean delivery; MoM = multiple of the median.

## Data Availability

All datasets are freely available at https://www.cdc.gov/nchs/data_access/Vitalstatsonline.htm (accessed on 2 December 2021).

## Supplementary Materials

The following supporting information can be downloaded at: https://www.mdpi.com/article/10.3390/jpm13071144/s1, Figure S1: Proportion of CD; Figure S2: Birth weight MoM time trend; Table S1: Complete multivariate logistic regression analysis.

## Abbreviations

ART: assisted reproduction technologies; BMI: body mass index; CD: cesarean delivery; CI.95: 95% confidence interval; ET: embryo transfer; FET: frozen embryo transfer; ICSI: intracytoplasmic sperm injection; IQR: interquartile range; IVF: in vitro fertilization; MoM: multiple of the median; OR: odds ratio; OST: other subfertility treatments; STROBE: Strengthening the Reporting of Observational Studies in Epidemiology; US: United States.
