# Supplementary material for: Assisted Reproductive Technology and Breech Delivery: A Nationwide Cohort Study in Singleton Pregnancies
Source: J Pers Med. 2023 Jul 16;13(7):1144. doi: 10.3390/jpm13071144 (PMC10381648; doi:10.3390/jpm13071144)

Figure S1- This plot shows the proportion of CD (yearly prevalence) in singleton pregnancies after OST, ART, and SC according to cephalic and breech presentation.

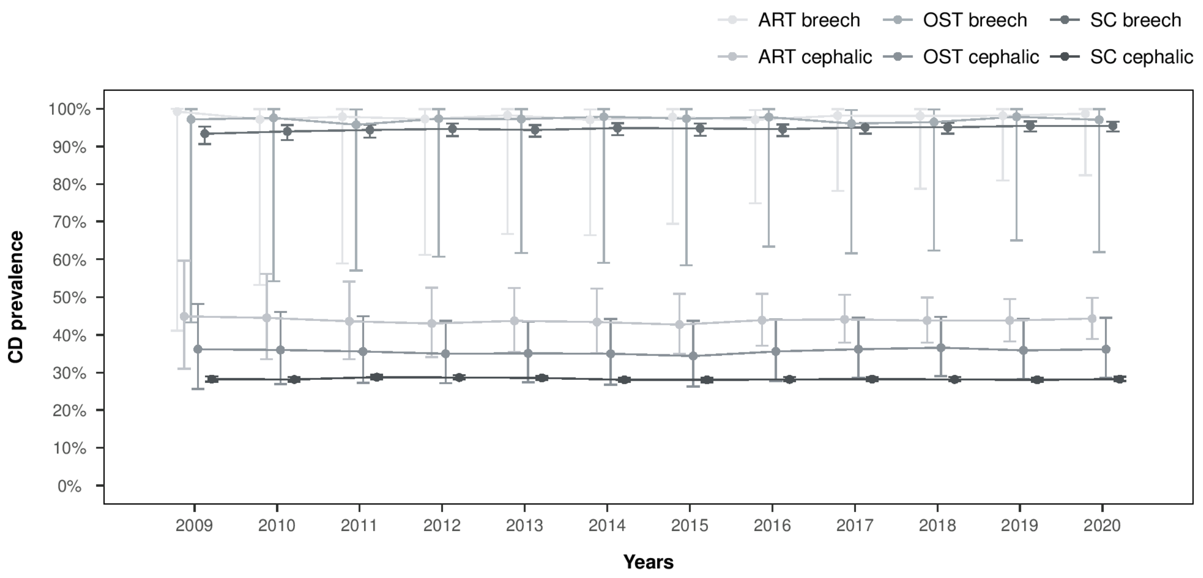

Supplement: Supplementary file 1 [file jpm-13-01144-s001.zip › Figure_S1_artus_v6.pdf]
