# Supplementary material for: Assisted Reproductive Technology and Breech Delivery: A Nationwide Cohort Study in Singleton Pregnancies
Source: J Pers Med. 2023 Jul 16;13(7):1144. doi: 10.3390/jpm13071144 (PMC10381648; doi:10.3390/jpm13071144)

**Figure S2**- This plot shows the birth weight MoM time trend in 154'395 OST pregnancies stratified per fetal presentation at delivery (breech vs. others) among the study years.

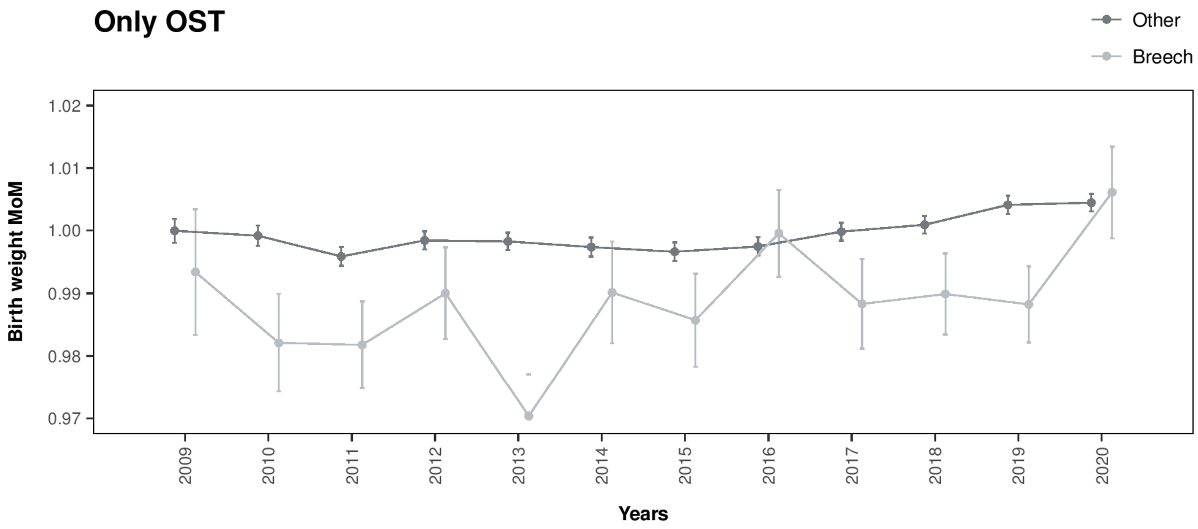

Supplement: Supplementary file 1 [file jpm-13-01144-s001.zip › Figure_S2_artus_v6.pdf]
