# Supplementary material for: Assisted Reproductive Technology and Breech Delivery: A Nationwide Cohort Study in Singleton Pregnancies
Source: J Pers Med. 2023 Jul 16;13(7):1144. doi: 10.3390/jpm13071144 (PMC10381648; doi:10.3390/jpm13071144)
Supplement: Supplementary file 1 [file jpm-13-01144-s001.zip › Table_S1_artus_v6.pdf]

Table S1- This table shows the complete multivariate logistic regression analysis (including interaction terms) that considers breech presentation at delivery as the dependent variable and the possible known risk factors as independent variables.

	OR (CI.95)(¶)	p(¶)
Factors associated with breech		
Maternal age >33 years	1.39 (1.38 - 1.41)	<0.001
Nulliparity	1.79 (1.78 - 1.81)	<0.001
Black (only) race	0.75 (0.74 - 0.76)	<0.001
Hispanic origin	0.81 (0.8 - 0.82)	<0.001
Tobacco smoke	1.18 (1.16 - 1.2)	<0.001
Previous CD	1.53 (1.51 - 1.55)	<0.001
Neonatal female sex	1.2 (1.19 - 1.21)	<0.001
Gestational age >40 weeks	0.41 (0.4 - 0.42)	<0.001
Birth weight (MoM)		
0.9-1.07 MoM		
<0.9 MoM	1.47 (1.46 - 1.49)	<0.001
>1.07 MoM	1.06 (1.05 - 1.07)	<0.001
OST	1.79 (1.71 - 1.87)	<0.001
ART	2.32 (2.23 - 2.41)	<0.001
Maternal age >33 years:Nulliparity	1.14 (1.13 - 1.15)	<0.001
Maternal age >33 years:Black (only) race	1.34 (1.32 - 1.36)	<0.001
Maternal age >33 years:Hispanic origin	1.22 (1.21 - 1.23)	<0.001
Maternal age >33 years:Tobacco smoke	1.12 (1.09 - 1.14)	<0.001
Maternal age >33 years:Neonatal female sex	0.95 (0.94 - 0.96)	<0.001
Maternal age >33 years:Gestational age >40 weeks	0.94 (0.93 - 0.96)	<0.001
Maternal age >33 years:Birth weight (MoM) <0.9 MoM	1 (0.99 - 1.01)	0.798
Maternal age >33 years:Birth weight (MoM) >1.07 MoM	0.98 (0.97 - 1)	0.006
Maternal age >33 years:OST	0.87 (0.83 - 0.91)	<0.001
Maternal age >33 years:ART	0.85 (0.81 - 0.88)	<0.001
Nulliparity:Black (only) race	0.72 (0.71 - 0.73)	<0.001
Nulliparity:Hispanic origin	0.85 (0.84 - 0.86)	<0.001
Nulliparity:Tobacco smoke	0.8 (0.79 - 0.82)	<0.001
Nulliparity:Neonatal female sex	1.03 (1.02 - 1.04)	<0.001
Nulliparity:Gestational age >40 weeks	0.86 (0.85 - 0.88)	<0.001
Nulliparity:Birth weight (MoM) <0.9 MoM	0.83 (0.82 - 0.84)	<0.001
Nulliparity:Birth weight (MoM) >1.07 MoM	1.01 (1 - 1.03)	0.028
Nulliparity:OST	0.76 (0.72 - 0.8)	<0.001
Nulliparity:ART	0.69 (0.67 - 0.72)	<0.001
Black (only) race:Hispanic origin	1.32 (1.29 - 1.35)	<0.001
Black (only) race:Tobacco smoke	0.92 (0.9 - 0.94)	<0.001
Black (only) race:Previous CD	0.98 (0.96 - 0.99)	0.007
Black (only) race:Neonatal female sex	0.94 (0.93 - 0.96)	<0.001
Black (only) race:Birth weight (MoM) <0.9 MoM	0.98 (0.97 - 1)	0.011
Black (only) race:Birth weight (MoM) >1.07 MoM	1.19 (1.17 - 1.21)	<0.001
Black (only) race:OST	1.18 (1.05 - 1.32)	0.004
Black (only) race:ART	1.26 (1.16 - 1.36)	<0.001
Hispanic origin:Tobacco smoke	1.11 (1.07 - 1.14)	<0.001
Hispanic origin:Previous CD	0.98 (0.96 - 0.99)	<0.001
Hispanic origin:Neonatal female sex	0.95 (0.94 - 0.96)	<0.001
Hispanic origin:Gestational age >40 weeks	1.18 (1.16 - 1.21)	<0.001
Hispanic origin:Birth weight (MoM) <0.9 MoM	0.98 (0.96 - 0.99)	<0.001
Hispanic origin:Birth weight (MoM) >1.07 MoM	1.13 (1.11 - 1.14)	<0.001
Hispanic origin:OST	1.24 (1.14 - 1.34)	<0.001
Hispanic origin:ART	1.1 (1.03 - 1.17)	0.004
Tobacco smoke:Previous CD	0.87 (0.85 - 0.89)	<0.001

Tobacco smoke:Neonatal female sex	1.02 (1 - 1.03)	0.03
Tobacco smoke:Gestational age >40 weeks	1.14 (1.1 - 1.17)	<0.001
Tobacco smoke:Birth weight (MoM) <0.9 MoM	0.97 (0.95 - 0.99)	<0.001
Tobacco smoke:Birth weight (MoM) >1.07 MoM	1.12 (1.1 - 1.14)	<0.001
Tobacco smoke:OST	0.8 (0.65 - 0.98)	0.035
Tobacco smoke:ART	0.62 (0.47 - 0.81)	<0.001
Previous CD:Neonatal female sex	0.97 (0.95 - 0.98)	<0.001
Previous CD:Gestational age >40 weeks	1.33 (1.3 - 1.36)	<0.001
Previous CD:Birth weight (MoM) <0.9 MoM	0.95 (0.94 - 0.96)	<0.001
Previous CD:Birth weight (MoM) >1.07 MoM	0.97 (0.96 - 0.99)	<0.001
Previous CD:OST	0.76 (0.71 - 0.83)	<0.001
Previous CD:ART	0.69 (0.65 - 0.72)	<0.001
Neonatal female sex:Birth weight (MoM) <0.9 MoM	0.99 (0.98 - 1)	0.062
Neonatal female sex:Birth weight (MoM) >1.07 MoM	0.96 (0.95 - 0.97)	<0.001
Gestational age >40 weeks:Birth weight (MoM) <0.9 MoM	1.13 (1.11 - 1.15)	<0.001
Gestational age >40 weeks:Birth weight (MoM) >1.07 MoM	0.92 (0.88 - 0.95)	<0.001
Gestational age >40 weeks:OST	0.84 (0.75 - 0.93)	<0.001

Missing values: (¶) 851640.
Acronyms: OST = other subfertility treatments; ART = assisted reproductive technologies; BMI = body mass index; CD = cesarean delivery; MoM = multiple of the median.
